# Supplementary material for: The interactome of intact mitochondria by cross-linking mass spectrometry provides evidence for coexisting respiratory supercomplexes
Source: Mol Cell Proteomics. 2017 Dec 8;17(2):216–32. doi: 10.1074/mcp.RA117.000470 (PMC5795388; doi:10.1074/mcp.RA117.000470)
Supplement: Supplemental Data [file supp_RA117.000470_133922_0_supp_23978_fzffwf.zip › spectra_annotation/mito_spectra_annotation/210-1-4-1-6-1.pdf]

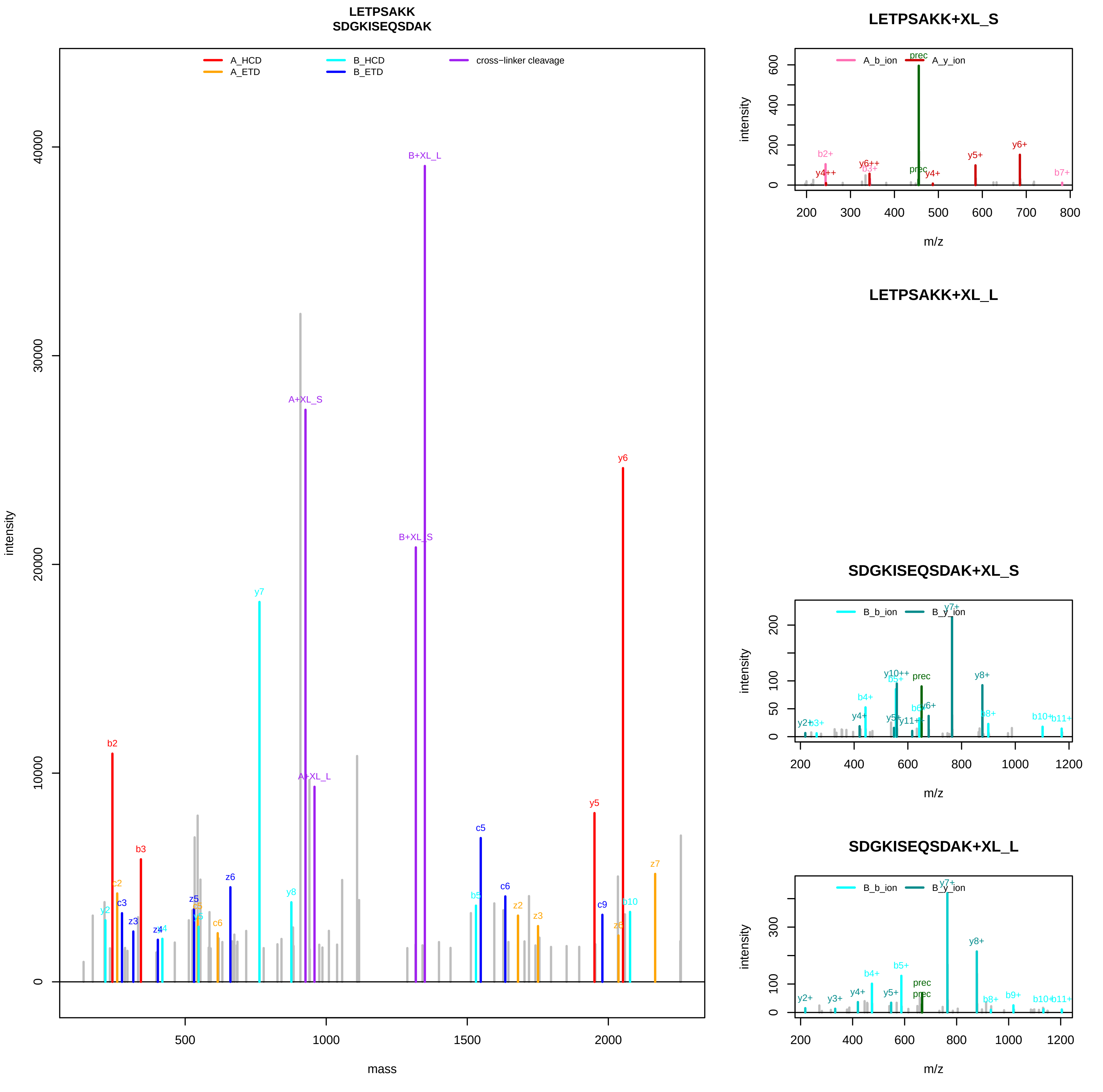
LETPSAKK
SDGKISEQSDAK
A_HCD
A_ETD
B_HCD
B_ETD
cross−linker cleavage
intensity
mass
B+XL_L
A+XL_S
B+XL_S
A+XL_L
y6
y5
y7
y8
b2
b3
c2
c3
z3
z4
z5
z6
c6
c5
c9
b5
b10
z2
z7
LETPSAKK+XL_S
A_b_ion
A_y_ion
prec
m/z
LETPSAKK+XL_L
SDGKISEQSDAK+XL_S
B_b_ion
B_y_ion
y7+
y8+
y10++
b5+
b4+
y4+
y6+
b8+
b10+
b11+
SDGKISEQSDAK+XL_L
y2+
y3+
y5+
b9+
